# Rehydration conditions for isolation of high quality RNA from the lichen *Lobaria pulmonaria*

**DOI:** 10.1186/1756-0500-7-442

**Published:** 2014-07-10

**Authors:** Jennifer A Doering, Vivian PW Miao, Michele D Piercey-Normore

**Affiliations:** 1 Department of Biological Sciences, University of Manitoba, 50 Sifton Road, Winnipeg, MB R3T 2 N2, Canada; 2 Department of Microbiology and Immunology, University of British Columbia, 2350 Health Sciences Mall, Vancouver, BC V6T 1Z3, Canada

**Keywords:** cDNA, Lichen, *Lobaria pulmonaria*, RNA quality, RNA quantity, RT-PCR, Total RNA

## Abstract

**Background:**

The poikilohydric nature of lichens enables them to survive repeated episodes of desiccation by utilizing water when it becomes available. During rehydration, RNA-degrading endonucleases may be released, reducing RNA quantity and quality. Re-generation of a steady-state condition where RNA quantity and quality no longer fluctuate establishes a framework for development of new hypotheses for future investigations. Using *Lobaria pulmonaria* as a model species, the objective of this study was to compare the effect of different rehydration conditions on the quantity and quality of RNA from the rehydrated thallus*.*

**Findings:**

Spectrophotometric measurements of total RNA and cDNA were performed for samples prepared from dry lichen or lichen after rehydration (0.5 h, 1 h, 2 h, 4 h or 24 h), with limited light and dark conditions, and at three temperatures (15°C, 20°C or 32°C) for some of these conditions. The results showed that rehydration of the thallus for 4 h at 20°C in light yielded the highest concentration and quality of RNA. A higher RNA concentration was obtained in light than in dark conditions, but the RNA quality was unaffected.

**Conclusions:**

This study suggests that allowance of 4 h for thallus rehydration should be adequate to ensure complete recovery of transcription. After 4 h at 20°C further studies can be carried out on the RNA in this model species.

## Background

Water scarcity limits survival for most vascular plants because they cannot tolerate extensive dehydration. Poikilohydric organisms such as lichens and mosses have no mechanism to prevent dehydration, but can recover from low cell water content without physiological damage. Recovery of metabolic activity, such as transcription, upon rehydration has been studied in the vascular resurrection plant and moss [1], and is of interest in lichens.

Lichens are symbiotic associations between a lichen-forming fungus (mycobiont) and a green alga and/or a cyanobacterium [2] (photobionts). *Lobaria pulmonaria* is a foliose (leaf-like) lichen found in old growth forests [3]. The mycobiont (also designated *L. pulmonaria*) in this lichen is accompanied by both a green algal, *Dictyochloropsis reticulata*[4] and a cyanobacterial, *Nostoc* sp. [5] symbiont. *L. pulmonaria* has been considered as a model species by some researchers [4,6] and has been extensively studied from the perspective of herbivory [7], population genetics and dispersal [4], physiology [8], conservation biology [9], and sexual reproduction [10,11].

Most lichen and bryophyte thalli will dehydrate and rehydrate as the humidity in the surrounding atmosphere changes. Poikilohydric responses coincide with changes in the cell structure, biochemistry and physiology, e.g. photosynthesis [12]. One of the early activities of metabolism upon restoration of cell function after desiccation is thought to be transcript formation [1], in part because synthesis of antioxidant proteins protects cells from the rapid intracellular release of reactive oxygen species after rehydration [12]. However, RNA-degrading endonucleases released during rehydration [13], may reduce RNA quantity and quality [14]. After the effects of rehydration reach a steady-state condition representative of the natural lichen, further experimentation provides the opportunity to investigate a variety of questions such as the ecological plasticity of the lichen by monitoring phenotypic, physiological or transcriptional metrics in response to different rehydration conditions. Re-generation of a steady-state condition is very useful in setting up a framework for development of new hypotheses for future investigations. While it has been shown that RNA can be retrieved from rehydrated *Cladonia rangiferina* thalli [15], that RNA-seq analyses have been performed on natural lichens [16,17] and a cDNA library has been produced from rehydrated samples of the moss, *Tortula ruralis*[1], the establishment of a steady-state condition after thallus rehydration has not been examined.

The objective of this study was to compare the effect of different rehydration conditions on the quantity and quality of RNA from the thallus of *L. pulmonaria,* using total RNA and cDNA by reverse transcription PCR and the A_260/280_ absorbance ratio.

## Methods

Naturally desiccated thalli of *Lobaria pulmonaria* were collected in British Columbia, Canada between November and December 2011, and kept dry and in the dark at −20°C until used for experiments. Two specimens (VM5172 and VM5174) were collected from very large branches (more than15 feet long) of maple (*Acer macrophylum*) thought to have broken from the canopy during autumn storms, no more than 2–3 months earlier along Yellow Point Lodge trail, near Ladysmith, southeastern Vancouver Island. Another specimen (VM5190) was removed from the bole of a standing tree at Cultus Lake, ~100 km east of the city of Vancouver. Small pieces were excised from each lichen specimen, trimmed to 70–100 mg dry weight (DW), and then either kept dry (control), or rehydrated in 600 μL of sterile distilled water under various temperature and light regimes for up to 24 h (Table 1). A Low Temperature Incubator (Fisher Scientific, Ottawa, Canada), a Gravity Convection Incubator (Precision Scientific, Chennai, India) and an Innova40 Incubator (New Brunswick Scientific, Enfield, USA) held at 15°C, 20°C, or 32°C, respectively, were used. Light was supplied by Grow Lamps (Vita Lite, Duro test, 20 W) at 60 μmol photons m^−2^ s^−1^ in the 20°C and 32°C chambers and at 6 μmol photons m^−2^ s^−1^ in the 15°C chamber.

**Table 1:** **Experimental design showing 13 rehydration, light, and temperature conditions for three specimens of *Lobaria pulmonaria***

**Treatment**	**Rehydration with water**	**Temp (°C)**	**Light**	**Rehydration time (hours)**	**No. replicates for VM5172**	**No. replicates for VM5174**	**No. replicates for VM5190**
1	no	20	yes	0	3	3	3
2	yes	20	yes	0.5	3	3	3
3	yes	20	yes	1	3	3	3
4	yes	20	yes	2	3	3	3
5	yes	20	yes	4	3	3	3
6	yes	20	yes	24	3	3	3
7	yes	20	no	1	3	3	3
8	yes	20	no	4	3	3	3
9	yes	32	yes	1	3	3	3
10	yes	32	yes	4	3	3	3
11	yes	15	yes	1	3	3	3
12	yes	15	yes	4	3	3	3
13	yes	15	yes	24	3	3	3

RNA was isolated from samples ground in a mortar and pestle with Trizol (Life Technologies Inc., Burlington, Canada), and initial yield and quality were estimated spectrophotometrically using a NanoDrop 2000/2000c (ThermoScientific, Ottawa, Canada). Removal of contaminating DNA was achieved with a DNase kit (Life Technologies Inc., Burlington, Ontario) and the absence of contaminating DNA was confirmed by gel electrophoresis in 1% agarose and by performing PCR for mtr SSU (below). RNA samples were re-measured on the NanoDrop 2000/2000 c and re-checked on a 1% agarose gel. A cDNA synthesis kit (ThermoScientific, Ottawa, Canada) was then used to synthesize cDNA from the cleaned RNA using oligo dT and random primers.

PCR reactions from cDNA were conducted in 20 μl with 0.4-2 μg template, 1× PCR Buffer (200 mM Tris–HCl, pH 8.4; 500 mM KCl**),** 0.1 μM each dNTP, 2.5 mM MgCl_2_, 0.4 mM each primer and 1 U Pfu DNA polymerase, and were performed in a Biometra T-gradient thermal cycler (Applied Biosystems, Foster City, USA). The taxon-specific PCR primers used to detect sequences representing the fungal (mtr SSU, mitochondrial small subunit rRNA), algal (*Lpu_27,* a simple sequence repeat) and cyanobacterial (*nifH,* nitrogenase) symbionts, were mtr SSU1/ mtr SSU2R [18], Lpu_27 F and R [19] and NiFH-F/NiFH-R [20], respectively. A modified touchdown program [21] was used for detecting *Lpu_27* in representative preparations: denaturing at 94°C 3 min, then 29 cycles of 94°C 30 s, 20 s annealing (56°C first 2 cycles, then 54°C for 27 cycles) and extension at 72°C 30 s, then a final extension at 60°C 10 min. The PCR program for *nifH* was: 94°C 5 min, then 35 cycles of 94°C 1 min, 48°C 1 min, 72°C 1 min 15 s, and finally 72°C 7 min. For the mtr SSU-based PCR saturation curve experiment, 20 μl aliquots from master mixes with or without cDNA template were delivered into PCR tubes and run in a PCR machine executing the following program: 25 cycles of 94°C 1 min, 54°C 30 s, and 72°C 1 min. A pair of tubes (one with template and the other a negative control) was removed after every 2 cycles beginning with cycle 8, resulting in 9 pairs representing different numbers of cycles from 8 to 24 cycles. All other mtr SSU PCR experiments using cDNA employed only 17 cycles of amplification. Lichen DNA samples (extracted from source thalli following published methods [22]) were used as controls for some PCR, and these reactions were also conducted in 20 μl mixtures, but consisted of 0.5-1 ng template, 0.2 mM each dNTP, 2 mM MgCl_2_, and 1 U of *Taq* in 1× buffer (Life Technologies Inc., Burlington, ON) and either 0.2 μM each primer (for *Lpu_27*) or 0.5 μM each primer (for *nifH*). These experiments were run on a T100 thermal cycler (BioRad), using the same respective programs (above). All amplified products were electrophoresed on a 1% agarose gel and compared with a 1 Kb Plus DNA Ladder (Life Technologies Inc. Burlington, ON) for size estimation.

Statistical analyses were performed in the software program R [23]. Because the data were non-normally distributed (indicated by Anderson-Darling test of normality and Levene’s test of equal variances), the Kruskal-Wallis test (non-parametric) was used to determine significant differences between temperatures, light, and rehydration times, and the Mann–Whitney test (non-parametric) was used to determine pairwise significant differences shown in the graphs.

## Findings

**Figure 1:**
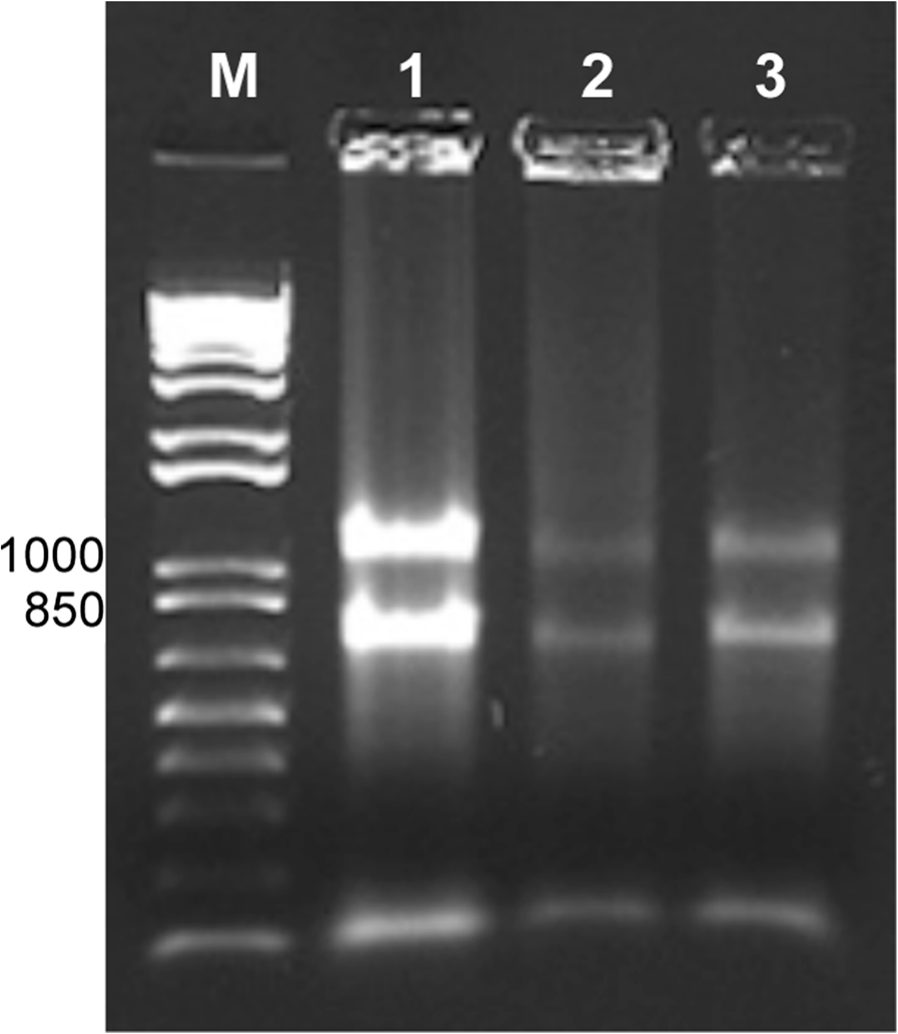
**Total RNA from *L. pulmonaria.*** Lanes 1–3 show representative preparations from specimens VM5172, VM5190, and VM5174, respectively. Lane M contains the 1 KB Plus DNA Ladder and some fragment lengths in base pairs.

Total RNA isolated from lichen thalli typically appeared as a smear (mRNA) with a sharp low molecular weight band (tRNA), and two bands representing 18S and 26S ribosomal RNAs (Figure 1). The yield of RNA was essentially the same from each of the three source specimens when all samples from each specimen (n = 39) were averaged together, *viz.* 2.9 ± 1.6 μg/gDW, 3.0 ± 2.0 μg/gDW and 3.0 ± 1.8 μg/gDW, respectively (Figure 1). The corresponding average RNA quality (A_260/280_) for VM5172 was 1.7 ± 0.2, for VM5190 was 1.7 ± 0.1; for VM5174 was 1.7 ± 0.1. However, the variance around the average quality differed among the three source specimens. Kruskal-Wallis (non-parametric ANOVA, p < 0.001) and Mann–Whitney (non-parametric t-test, p < 0.050 for comparison between VM5172 and VM5190; p < 0.001 for comparison between VM5174 and VM5190; p > 0.050 for the comparison between VM5172 and VM5174) tests showed that RNA quality of VM5190 was significantly higher than that of VM5174 and VM5172, but that there was no difference between the latter.

**Figure 2:**
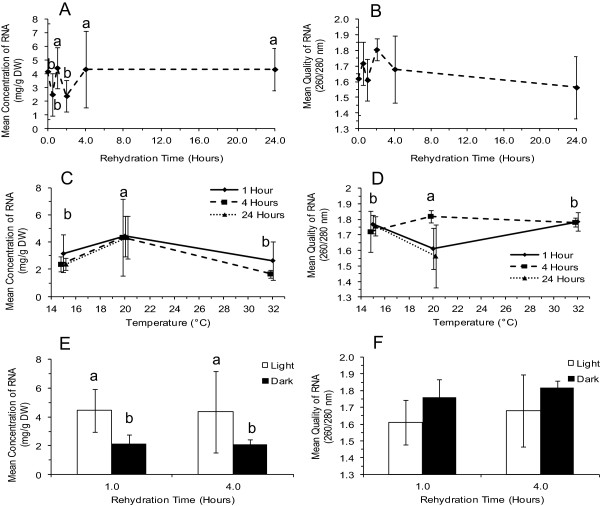
**The effect of rehydration conditions on RNA concentration (mg/gDW) and quality (A**_**260/280**_**). A**, **B**. Effect of rehydration time at 20°C in the light. **C**, **D**. Effect of temperature for rehydration times of 1 h, 4 h, and 24 h in the light. **E**, **F**. Effect of light at 20°C for rehydration times of 1 h and 4 h. Means and error bars representing standard deviations are shown. Data are offset to show all points, n = 9. Different lowercase letters indicate significantly different results; figures without lowercase letters show results that are not significantly different.

Significantly more RNA was recovered from samples rehydrated at 20°C for 1 h, 4 h, and 24 h than from those treated for 0.5 h and 2 h (Figure 2A), but there was overall no difference in RNA purity, as assessed by A_260/280_ ratio (Figure 2B). Incubation at 20°C was more productive than at either higher (32°C) or lower (15°C) temperatures (Figure 2C) and after 4 h rehydration, gave higher quality RNA (Figure 2D). Rehydration of lichen thalli in the light resulted in a higher yield of RNA (Figure 2E) without affecting quality (Figure 2F).

**Figure 3:**
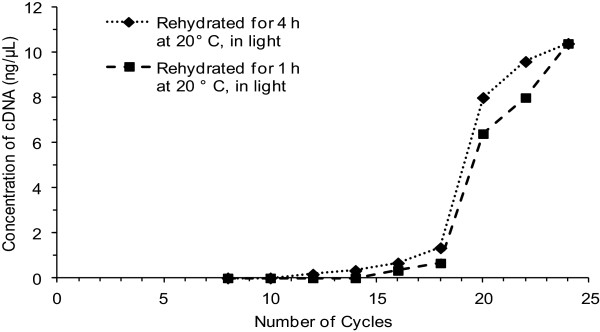
Saturation curves for rehydration conditions showing the concentration of cDNA and number of PCR cycles.

**Figure 4:**
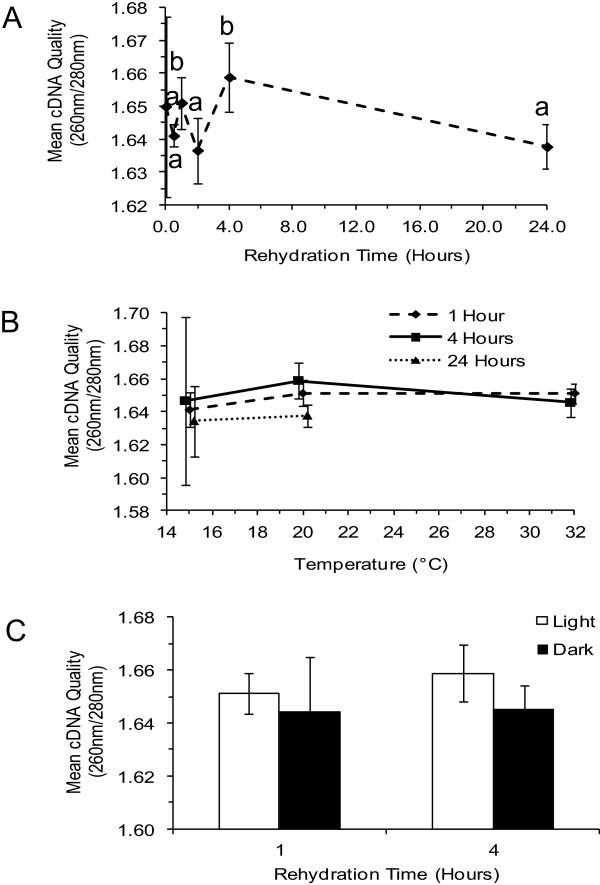
**The effect of rehydration conditions on cDNA quality (A**_**260/280**_**). A**. Effect of rehydration time (hours) at 20°C in the light. **B**. Effect of temperature for rehydration times of 1 h, 4 h, and 24 h in the light. **C**. Effect of light for rehydration times of 1 h and 4 h at 20°C. Error bars represent standard deviations of three replicates. Different lowercase letters indicate significantly different results; figures without lowercase letters show results that are not significantly different.

Reverse transcription PCR (RT-PCR) was also used to assess the quality of total *L. pulmonaria* RNA. Tests for taxon-specific genetic markers, mtr SSU, *Lpu_27*, and *nifH*, on cDNA synthesized with both oligo dT and random primers indicated that the mycobiont as well as both photobionts of *L. pulmonaria* were all represented (data not shown). Using mtr SSU as the mycobiont reference gene, trials with increasing numbers of amplification cycles were conducted and saturation of the PCR curve was observed between 17 and 21 cycles (Figure 3). This allowed standardization of RT-PCR tests at 17 cycles for a sensitive measure of original RNA quality based on amplicon accumulation. As found earlier by direct spectroscopic assessment of total RNA, the best results from RT-PCR were associated with samples rehydrated for 1 h and 4 h durations (Figure 4A) and 20°C (Figure 4B); again, there was no significant influence of light during incubation (Figure 4C).

## Discussion

The amount of RNA isolated, which was normalized for thallus dry weight, was smaller in this study than that in a study on *Cladonia rangiferina*[15], but whether this is of significance is unclear as the composition and morphology of the two lichens are very different. The very small amounts of starting material used here may be a factor, as the percentage loss during mortar grinding may be proportionately larger. Overall, the key result is that RNA of sufficient quantity and quality can be recovered to detect all symbionts, indicating that future studies will be possible even where biomass may be limited. The present work suggests that a wide variation in yield (standard deviation of 55% to 66% of the total RNA was calculated over all conditions) might be expected when using the Trizol method, and that it is accompanied by an inverse relationship between RNA quality and quantity (Figure 2). This may be generally applicable, as the C*. rangiferina* study, which compared several methods, also reported standard deviations of 26% to 72% on the average yield (ca Table 1[15]) and opposing trends between RNA quality and quantity.

The significant variation in RNA quality between thalli may, on first principles, be attributable to one or more variables such as different intrinsic metabolic rates of each thallus as a result of age, reproduction, stress, pollution, etc., or the dehydration and storage conditions that affect the revival capacity of the thallus [24] and therefore transcription. Interestingly, RNA quality was more similar between the two specimens from one locale (VM5172 and VM5174) than either compared to VM5190, from another locale. The environmental conditions in which each thallus was collected would provide a different acclimation process exposing each thallus to different levels of light and temperature regimes [6]. Therefore differences would be expected among thalli given the wide distribution and variation in habitat of the species [25]. *Nostoc* has been shown to have a significant increase in photosynthesis and gene activity upon rehydration [26]. The low variation in RNA concentration at 20°C (Figure 3) is reasonable since the optimal temperature for lichen growth and metabolism is thought to be 18°C to 20°C [2] and other studies also used rehydration temperatures between 18°C to 20°C [1,15]. In general, temperatures lower than this may be less optimal for lichen-forming algal or cyanobacterial photosynthesis and temperatures higher than this may damage the photosynthetic partner within the lichen, and presumably RNA transcription.

While the 1 h, 4 h, and 24 h rehydration periods at 20°C in the light provided the highest concentration of RNA, and 4 h provided the highest quality of RNA, the quantity and quality fluctuated within the first 4 h of testing. These fluctuations may reflect a combination of time needed to revive the thallus, plus another period for acclimation to ambient conditions. Photosynthesis and metabolism have been reported to begin within minutes after rehydration in the lichen, *Ramalina lacera*[27], but acclimation to ambient conditions may take longer periods of time, e.g. to fully activate enzymes. Ferrar and Smith [28] and [1] showed that revival of a lichen and a moss occurred within 2 h of rehydration. Here, 4 h appear to provide an adequate period for the thallus to reach a steady state, as there was either no increase, or a reduction, in the quantity and quality of RNA and cDNA at 24 h. Limitation of the amount of light to 1 h instead of 4 h may be beneficial since high light has been shown to cause the thallus temperature to increase and damage the thallus even when dry [6]. The higher concentration of RNA in light may be explained if genes for light activated photosynthetic enzymes were transcribed, producing a larger amount of RNA immediately upon rehydration, as has been observed in *R. lacera*[27] and in a poikilohydric moss [1].

## Conclusion

A steady-state condition that may be representative of the natural lichen is attained by *L. pulmonaria* thalli 4 h after rehydration, as determined by direct assessment of total RNA as well as by examination of cDNA quality. Such knowledge simplifies sample collection and *in situ* processing, allowing much greater flexibility for storage and transport of specimens without use of specialized reagents to assure RNA integrity. Experimentation to further our understanding of this model organism and other poikilohydric lichens may be pursued with some confidence that steady state conditions can be reached.

## Abbreviations

cDNA: Complementary DNA; DW: Dry weight; *nifH*: Nitrogenase gene; mtr SSU: Mitochondrial small subunit gene; *Lpu_27*: A simple sequence repeat locus; A_260/280_: Ratio of absorbance at 260 nm and 280 nm.

## Competing interests

The authors declare they have no competing interests.

## Authors’ contributions

JD contributed to study design, carried out all lab work and drafted the manuscript. VM collected the specimens, provided critical advice on the analysis and interpretation of data, and made significant revisions to the manuscript. MN provided funding, designed the study, provided advice and training to JD on the molecular biology, and made critical revisions of the manuscript. All authors have given final approval of the manuscript.
